# Editorial: Itch treatments

**DOI:** 10.3389/fmed.2024.1373702

**Published:** 2024-02-16

**Authors:** Martin Steinhoff, Shawn Kwatra, Laurent Misery

**Affiliations:** ^1^Department of Dermatology and Venereology, Hamad Medical Corporation, Doha, Qatar; ^2^Translational Research Institute, Academic Health System, Hamad Medical Corporation, Doha, Qatar; ^3^Dermatology Institute, Academic Health System, Hamad Medical Corporation, Doha, Qatar; ^4^Department of Medicine, Weill Cornell Medicine-Qatar, Ar-Rayyan, Qatar; ^5^College of Medicine, Qatar University, Doha, Qatar; ^6^School of Life Sciences, Hamad-bin-Khalifa University, Doha, Qatar; ^7^Department of Dermatology, Weill Cornell Medicine, New York, NY, United States; ^8^Department of Dermatology, Johns Hopkins University, Baltimore, MD, United States; ^9^Department of Dermatology, Venereology, and Allergology, University Hospital of Brest, Brest, France; ^10^French Expert Centre on Pruritus, Brest, France; ^11^Univ. Brest, LIEN, Brest, France

**Keywords:** itch, atopic dermatitis, pathophysiology, cytokine, JAK, therapy, biologic AD, DM

## Introduction

Similar to pain, pruritus (itch) is a very common somato-sensory sensation that almost everyone experiences in their lifetime. Once dysregulated, pruritus can become chronic and debilitating, significantly affecting a patient's and their family's quality of life (1). Molecular biology and translational research have helped to better identify and understand the molecular mechanisms of the various pathways of histamine-dependent and -independent itch and supported the development of new treatment options for the different subtypes of pruritic diseases, whether inflammatory or non-inflammatory, neurologic or neuropathic, for example (2–6). Furthermore, to identify the cellular mechanisms that differentiate and specify the different itch pathways in the various pruritic diseases is critical (7). The treatment options for CP span a number of topical and systemic medications as well as phototherapy, depending on the underlying disease, age, and comorbidities (8, 9). Even in pruritus of unknown origin, the anti-IL-4 receptor-a antibody dupilumab has been shown to be effective, at least in a case report, suggesting that cytokines and microinflammation play a role in presumptive “non-inflammatory” conditions to a larger extent than anticipated (1, 6). However, neuro-modulatory agents are still necessary in various pruritic diseases, especially to treat neuropathic pruritus (2, 5) or prurigo (7). Here, extensive translational and clinical studies are necessary to stratify the best treatment.

## New aspects about the pathophysiology, diagnosis, and management of pruritus and prurigo

This Research Topic addresses various aspects about the pathophysiology, diagnostic workup, and management of various pruritic diseases including prurigo.

One important aspect in some pruritic diseases and prurigo is that of small fiber neuropathies (2, 5). In this Research Topic, Fouchard et al. have demonstrated, in an observational retrospective monocentric case–control study of patients with small-fiber neuropathies (SFNs), a potential positive correlation to smoking (active and past) and vitamin D deficiency. Here, further studies are warranted.

The molecular mediators, receptors, and pathways that regulate pruritus in atopic dermatitis and prurigo are partly understood. The fact that anti-cytokine therapy (e.g., dupilumab) and JAK inhibitors (JAK1, JAK2) are capable of alleviating itch in atopic dermatitis and - beyond – prurigo, pruritus of unknown origin, and chronic spontaneous urticaria, for example, indicates that cytokines and JAK/STAT pathways are involved in various pruritic diseases that do not have a marked inflammatory infiltrate (7). As to whether microinflammatory circuits and/or direct inactivation of neuronal cytokine receptors and/or intraneuronal JAK pathways in so-called non-inflammatory pruritic diseases play a role (e.g., uremic pruritus, prurigo) is unknown and needs to be further investigated.

Along that line, the mechanistic role of fibroblasts in AD and prurigo are still poorly understood, leading to fibrotic changes in the latter. Using RNA sequencing, transcriptomics, and imaging technologies, in this Research Topic, Deng et al. have investigated the similarities and differences of fibroblast function in AD compared to PN patients. They have showed that PN skin expressed higher levels of certain cytokines (including IL-1a and -b, OSM), neurotrophins (NGF), growth factors (IGF-1), and various chemokines, for example. NGF immunoreactivity was found to be higher in lesional PN compared to non-lesional and AD. More mechanistic studies are warranted to clarify the critical molecular pathways and thus therapeutic targets for PN and its impact on the regulation of extracellular matrix proteins, dermal nerve growth factor, cytokines, and chemokines as compared to AD.

An important mechanistic study with respect to understanding itch mechanisms is – given the obvious importance of cytokines in some diseases - to which extent neuromediators/peptides play a role as targets for the treatment of pruritic diseases and prurigo, e.g., in recalcitrant chronic cases of neuropathic and central itch. One overview article by Steinhoff et al. has demonstrates that – beside cytokines – neuromediators play a role as inducers of itch and neuroinflammation in a bidirectional way: one the one hand, neuropeptides such as those released by sensory nerve endings induce the production and release of neuropeptides such as endothelin-1 or cytokines such as TSLP (10), which, in turn, activate their high-affinity receptors on nerve endings to induce itch. Simultaneously, cytokines such as IL-4, -13, or -31 stimulate the release of neuropeptides that aggravate itch by releasing more itch mediators from keratinocytes (6). Thus, besides cytokines and JAK-inhibitors, neuromediators are also targets to treat pruritus and prurigo.

Another important aspect in in this Research Topic is the different pathophysiological pathways in specific diseases such as urticaria, atopic dermatitis, or bullous pemphigoid, for example. Verifying these pathways will help to develop novel therapies tailored to the specific pruritic diseases. Here, Rosenthal et al. have shown, in a retrospective study, that sodium cromoglycate significantly improved pruritus in BP patients when treated for at least 4 weeks. Prospective multicenter studies will be helpful to validate this observation.

In recent years, we have observed a substantial development for topical and systemic medications for the treatment of atopic dermatitis. Here, Rodriguez-Le Roy et al. have given an excellent systematic overview of the efficacy of classical and novel topicals and systemics to treat pruritus in AD patients conducted until December 2021, including 56 studies. However, we need to take into consideration, as a limitation, that the comparison of atopic dermatitis-related medications with respect to pruritus efficacy derives from using different assessment tools; here, an international standardization of assessments would be beneficial for both dermatologists and patients to choose and receive the best medication from the beginning.

One of the thus far largest regional studies about the correlation of urticaria and associated symptoms has been published in this Research Topic. Here, Wang X. et al. have shown in a multicenter, questionnaire-based study with more than 1,700 patients that delayed treatment success positively correlated with itch severity. While chronic urticaria can be controlled in most of the cases, we still need more epidemiological data at the global level and better therapeutic stratification of the various subtypes (e.g., which patients will not respond to the common guideline regimen (four antihistamines plus anti-IgE antibody) and thus may benefit from cyclosporine A treatment from beginning without delay). Similarly, the treatment of pressure urticaria is often still a therapeutic challenge.

Finally, there is still a big gap in our understanding of adjuvant therapies including acupuncture, hypnosis, or relaxation techniques, for example, for the treatment of pruritic diseases and prurigo. In this Research Topic, we find a systemic review and meta-analysis summarizing the efficacy of acupoint stimulation as a treatment for uremic pruritus (Lu et al.). Along that line, Li et al. have summarized the impact of acupuncture for gastrointestinal urticaria in a systematic review and meta-analysis. To which extent the results can be extrapolated to other adjuvant neuromodulatory or cognitive therapies as mono- or combined therapy, or other diseases, will be an exciting question for future research.

## Summary and open questions

This Research Topic highlights some novel pathophysiological and therapeutic aspects of pruritus and prurigo and may lead to new avenues to expand our therapeutic armamentarium to control chronic pruritus or prurigo in the future.

First, our knowledge about the pathophysiological mechanisms that are critical in pruritus or prurigo is still sparse. One of the interesting aspects will be to learn more about the role of the fibroblast in atopic dermatitis and prurigo, which leads to fibrotic changes in the latter. Another important aspect in in this Research Topic is the different pathophysiological pathways in specific diseases such as atopic dermatitis, different forms of prurigo, urticaria, or bullous pemphigoid. Verifying these pathways will help to develop novel therapies tailored to specific pruritic diseases. Another exiting question addressed is to better understand what the similarities and differences in the pathobiology of atopic dermatitis and prurigo are. Finally, we need deeper knowledge about the different optimal treatment options among the various inflammatory and systemic or neuropathic pruritic diseases. Consequently, one systematic literature review and meta-analysis has analyzed the efficacy of topical and systemic treatments for atopic dermatitis in the context of pruritus.

## Author contributions

MS: Conceptualization, Data curation, Formal analysis, Investigation, Methodology, Supervision, Validation, Writing—original draft, Writing—review & editing. SK: Data curation, Formal analysis, Investigation, Writing—original draft, Writing—review & editing. LM: Conceptualization, Data curation, Investigation, Methodology, Validation, Writing—original draft, Writing—review & editing.
